# Interferon β-1a (IFNβ-1a) in COVID-19 patients (INTERCOP): study protocol for a randomized controlled trial

**DOI:** 10.1186/s13063-020-04864-4

**Published:** 2020-11-23

**Authors:** Emanuele Bosi, Carlo Bosi, Patrizia Rovere Querini, Nicasio Mancini, Giliola Calori, Annalisa Ruggeri, Cecilia Canzonieri, Luciano Callegaro, Massimo Clementi, Francesco De Cobelli, Massimo Filippi, Marco Bregni

**Affiliations:** 1 Unit of Internal Medicine, IRCCS Ospedale San Raffaele, Milan, Italy; 2 Vita-Salute San Raffaele University, Milan, Italy; 3 University of Milan Medical School, Milan, Italy; 4 Unit of Virology, IRCCS Ospedale San Raffaele, Milan, Italy; 5 Clinical Trial Center, IRCCS Ospedale San Raffaele, Milan, Italy; 6 Unit of Radiology, IRCCS Ospedale San Raffaele, Milan, Italy; 7 Unit of Neurology, IRCCS Ospedale San Raffaele, Milan, Italy

**Keywords:** IFNβ-1a, COVID-19, SARS-CoV-2

## Abstract

**Background:**

Pharmacological therapies of proven efficacy in coronavirus disease 2019 (COVID-19) are still lacking. We have identified IFNβ-1a as the most promising drug to be repurposed for COVID-19. The rationale relies on the evidence of IFNβ anti-viral activity in vitro against SARS-CoV-2 and animal models resembling SARS-CoV-2 infection and on a recent clinical trial where IFNβ was indicated as the key component of a successful therapeutic combination.

**Methods:**

This is a randomized, controlled, open-label, monocentric, phase II trial (INTERCOP trial). One hundred twenty-six patients with positive swab detection of SARS-CoV-2, radiological signs of pneumonia, and mild-to-moderate disease will be randomized 2:1 to IFNβ-1a in addition to standard of care vs standard of care alone. No other anti-viral drugs will be used as part of the regimens, both in the control and the intervention arms. IFNβ-1a will be administered subcutaneously at the dose of 44 mcg (equivalent to 12 million international units) three times per week, at least 48 h apart, for a total of 2 weeks. The primary outcome is the time to negative conversion of SARS-CoV-2 nasopharyngeal swabs. Secondary outcomes include improvement or worsening in a clinical severity score measured on a 7-point ordinal scale (including transfer to intensive care unit and death), oxygen- and ventilator-free days, mortality, changes in pulmonary computed tomography severity score, hospital stay duration, reduction of viral load measured on nasopharyngeal swabs, number of serious adverse events, and changes in biochemical markers of organ dysfunction. Exploratory outcomes include blood cell counts, cytokine and inflammatory profile, peripheral mRNA expression profiles of interferon-stimulated genes, and antibodies to SARS-CoV-2 and to IFNβ-1a. INTERCOP is the first study to specifically investigate the clinical benefits of IFNβ-1a in COVID-19 patients.

**Discussion:**

Potential implications of this trial are multifaceted: should the primary outcome be fulfilled and the treatment be safe, one may envisage that IFNβ-1a be used to reduce the infectivity of patients with mild-to moderate disease. In case IFNβ-1a reduced the duration of hospital stay and/or ameliorated the clinical status, it may become a cornerstone of COVID-19 treatment.

**Trial registration:**

EudraCT 2020-002458-25. Registered on May 11, 2020

ClinicalTrials.gov Identifier: NCT04449380

## Administrative information

**Table:** 

Title{1}	Randomized, controlled, open label, phase 2 clinical trial of Interferon β-1a (IFNβ-1a) in COVID-19 patients (INTERCOP)
Trial registration {2a and 2b}.	EudraCT 2020-002458-25, registered on May 11, 2020 ClinicalTrials.gov Identifier: NCT04449380
Protocol version {3}	Issue Date: 14 May 2020 Protocol Number: 0
Funding {4}	The sponsor of the trial is IRCCS Ospedale San Raffaele, Milan, Italy. A contract between IRCCS Ospedale San Raffaele (via Olgettina, 60 – Milan, Italy) and Merck Serono S.p.A. (Manufacturer and Marketing Authorization Holder) assigned a grant to cover the costs of drug (Rebif®) supply and delivery.
Author details {5a}	Emanuele Bosi^1,2^, Carlo Bosi^3^, Patrizia Rovere Querini^1,2^, Nicasio Mancini^2,4^, Giliola Calori^5^, Annalisa Ruggeri^5^, Cecilia Canzonieri^5^, Luciano Callegaro^5^, Massimo Clementi^2,4^, Francesco De Cobelli^2,6^, Massimo Filippi^2,7^, Marco Bregni^5^ ^1^Unit of Internal Medicine, IRCCS Ospedale San Raffaele, Milan, Italy; ^2^Vita-Salute San Raffaele University, Milan, Italy; ^3^University of Milan Medical School, Milan, Italy; ^4^Unit of Virology, ^5^Clinical Trial Center, ^6^Unit of Radiology and ^7^Unit of Neurology, IRCCS Ospedale San Raffaele, Milan, Italy
Name and contact information for the trial sponsor {5b}	Trial Sponsor: Prof. Emanuele Bosi – IRCCS Ospedale San Raffaele Address: Via Olgettina, 60 - 20132 Milan – ITALY Email: bosi.emanuele@hsr.it
Role of sponsor {5c}	This is an investigator-initiated trial. IRCCS Ospedale San Raffaele and Merck Serono have no role on the design, collection, management, analysis and interpretation of data. Both entities do not have authority over writing or submitting the report.

## Introduction

### Background and rationale {6a}

Type I interferons (interferon alpha and beta, IFNα and IFNβ) are cytokines with antiviral and immuno-modulatory effects. By virtue of these properties, IFNα was used in the past as a treatment for chronic Hepatitis-C [1], while IFNβ is one of the reference treatments in multiple sclerosis in two therapeutic formulations: IFNβ-1a and IFNβ-1b [2].

Previous investigation in severe acute respiratory syndrome coronavirus (SARS-CoV) [3] and Middle East respiratory syndrome coronavirus (MERS-CoV) [4] infections indicated that IFNβ might be a valid candidate to treat COVID-19.

While there are no clinical trials in SARS, a study testing IFNβ-1b in MERS patients is currently ongoing and results are expected by December 2020 (MIRACLE trial, NCT00419562).

In a mouse model of MERS, IFNβ-1b ameliorated the pulmonary function of infected mice when administered in therapeutic settings at a dose mirroring that currently used in the MIRACLE trial, although no changes in signs of acute lung injury were observed [5]. Moreover, IFNβ-1b was efficacious in the nonhuman primate common marmoset model of MERS [6].

A recent study of SARS-CoV-2 infection has reported that type III and type-I interferons are downregulated both in SARS-CoV-2 infected cells and in the lung tissue of patients deceased with COVID-19 [7], suggesting a pathogenic mechanism similar to that hypothesized for SARS-CoV and MERS-CoV [8]. Furthermore, some from our group have recently shown that IFNβ inhibits replication of SARS-CoV-2 in vitro [9]*.*

The unprecedented emergency of the COVID-19 pandemic, with no available drugs of proven efficacy, provided a compelling reason to repurpose drugs already marketed with other indications for treating SARS-CoV-2 infection. Among these, IFNβ looked immediately attractive and some clinical trials were started, including the World Health Organization (WHO) sponsored SOLIDARITY trial program, in which IFNβ is being tested in combination with lopinavir/ritonavir [10]. Furthermore, an open label, randomized trial showed that IFNβ-1b, in addition to lopinavir-ritonavir and ribavirin, was superior to lopinavir-ritonavir alone in shortening the time to negative conversion of nasal swabs for SARS-CoV-2, alleviating symptoms and reducing the duration of hospital stay [11]. The findings of this study indicate the urgent need of trials specifically addressed to test IFNβ efficacy in COVID-19 patients [12].

Timing of intervention might be crucial when it comes to COVID-19 immunomodulation. Since the clinical manifestations of SARS-COV-2 infection range from mild symptoms (fever, malaise, cough) to bilateral pneumonia leading to progressive respiratory failure, it is likely that a compound with both antiviral and immunomodulatory effects such as IFNβ would be most effective in the earliest stages of the disease. Nonetheless, IFNβ-1a is likely to be safe also in the case of progression to ARDS, as shown in a recent trial [13].

### Objectives {7}

The purpose of this randomized clinical trial is to test the efficacy and safety of IFNβ-1a in COVID-19 patients at early stages of SARS-COV-2 infection. Patients identified as suitable for the enrolment are those symptomatic at the time of diagnosis, with mild-to-moderate radiologic signs of pneumonia and no acute respiratory distress syndrome (ARDS).

### Trial design {8}

This is a superiority, randomized, controlled, open label, phase 2 clinical trial of IFNβ-1a in COVID-19 patients (INTERCOP). Recruited patients will be randomized 2:1 to either of two parallel arms: the intervention or the control arm.

## Methods: participants, interventions, and outcomes

### Study setting {9}

This clinical trial will enroll patients at the IRCCS San Raffaele Hospital, an academic hospital part of the Gruppo San Donato (GSD) hospital network located in Northern Italy. Patients admitted to other hospitals of GSD will be offered to participate to the trial: if they are admitted to the trial, they will be transferred to IRCCS San Raffaele.

### Eligibility criteria {10}

Participants will be patients aged 18 years or greater, diagnosed with COVID-19 (positive swab RT-PCR detection of SARS-CoV-2 and pneumonia diagnosed with computed tomography (CT)), and with a clinical status of 3, 4, or 5 on a 7-point ordinal scale, consisting of the following: (1) not hospitalized, with resumption of normal activities; (2) not hospitalized, but unable to resume normal activities; (3) hospitalized, not requiring supplemental oxygen; (4) hospitalized, requiring supplemental oxygen; (5) hospitalized, requiring high-flow oxygen therapy, non-invasive mechanical ventilation, or both; (6) hospitalized, requiring extracorporeal membrane oxygenation (ECMO), invasive mechanical ventilation, or both; and (7) death. Exclusion criteria include clinical status defined as category 1, 2, or 6 on the aforementioned 7-point ordinal scale, known allergy or hypersensitivity to IFNβ-1a or IFNβ-1b; presence of severe concomitant illnesses and/or medical conditions that in the physician opinion will not allow participation to the study; pregnant or lactating females; history of major depression disorder or suicidal attempt or suicidal ideation; and spontaneous blood ALT or AST levels > 5 times the upper limit of normal. Starting from the signature of the informed consent, the patient will be considered enrolled in the clinical trial and subject to randomization. Interventions will be performed by the San Raffaele Hospital physicians assigned to the COVID-19 units.

### Who will take informed consent? {26a}

Every patient will be informed about the modalities of the clinical study in accordance with the patient informed consent document, both in writing and verbally by the investigating physician. The informing physician and the patient must each personally sign and date the informed consent form with a declaration on data privacy. Every informed consent will be part of the investigator’s file and retained with it. The patient will retain a copy of the patient informed consent.

### Additional consent provisions for collection and use of participant data and biological specimens {26b}

In addition to consent to the trial, patients will be asked to sign a second informed consent for collection samples for mechanistic studies and the COVID-19 biobank project (COVID-BioB- ClinicalTrials.gov NCT04318366).

### Interventions

#### Explanation for the choice of comparators {6b}

Patients randomized to the intervention arm will receive IFNβ-1a in addition to standard of care, while patients of the control arm will receive standard of care only. The standard of care for COVID-19 patients at San Raffaele Hospital includes any pharmacological (e.g., antibiotics) and non-pharmacological (e.g., oxygen, ventilation) treatments prescribed on clinical grounds. In consideration of the emerging role of thrombosis in COVID-19 pathogenesis [14, 15] and in the absence of apparent indications of harm [16], heparin will be part of background therapy in all patients. In the absence of clear efficacy data in patients with mild-to-moderate disease [17, 18], no additional antiviral treatments (e.g., remdesivir, hydroxychloroquine, lopinavir/ritonavir) will be administered in either the experimental or the control arms. In case of ongoing therapies with hydroxychloroquine and/or lopinavir/ritonavir (e.g., as a general practitioner’s prescription), these drugs are discontinued prior to admission to the trial.

#### Intervention description {11a}

##### Intervention arm

IFNβ-1a Rebif® (Merck Europe BV, Amsterdam, The Netherlands) will be used as the investigational drug. IFNβ-1a will be administered subcutaneously at a dose of 44 mcg (equivalent to 12 million international units), three times per week at least 48 h apart, for a total of 2 weeks. All patients will receive a total dose of 264 mcg (72 million international units) under physician control; no dose adjustments are planned. This dose corresponds to that recommended in relapsing-remitting multiple sclerosis [19, 20]. There will not be gradual dose escalation in order to maximize drug effect in the quickest time.

##### Control arm

Any pharmacological (e.g., antibiotics) and non-pharmacological (e.g., oxygen, ventilation) treatments prescribed on clinical grounds.

#### Criteria for discontinuing or modifying allocated interventions {11b}

Patients may drop out of the clinical study at any time without stating reasons. This will not have any negative consequences for the patient’s further treatment.

Possible reasons for withdrawal are:

- Withdrawal of consent.
- Any clinical condition that in the investigator’s opinion could become dangerous for the patient and prevent the good conduction of the clinical trial.
- Protocol violation that could compromise the quality of study data.
- Lack of co-operation/compliance of the patient.
- Occurrence of new diseases that could influence the treatment efficacy, for which the study medication is contraindicated or that are treated with a medication that is not permitted as a concomitant medication.
- Lack of experimental treatment administration due to failure to release the Medicinal Product lot.
- Lost to the follow-up.

The patients may withdraw from the study, if they decide to do so, at any time and for any reason.

Patients who have been withdrawn from the study cannot be re-included in the trial.

#### Strategies to improve adherence to interventions {11c}

Since the study drug is provided by the Hospital Pharmacy and has to be administered subcutaneously, the study nurse and medical staff involved in the trial will have direct control on drug administration.

#### Relevant concomitant care permitted or prohibited during the trial {11d}

Hydroxychloroquine, lopinavir/ritonavir, and remdesivir will not be administered as part of the standard therapeutic regimen, either in the intervention or in the control arms. Given the promising results of remdesivir in patients with severe disease [17], clinicians will be offered to administer remdesivir to patients with a clinical status of 6 on the 7-point ordinal scale.

### Provisions for post-trial care {30}

All patients hospitalized at San Raffaele with a diagnosis of COVID-19 are entering a post-discharge monitoring clinical program, independently on their participation to clinical trials. The purpose of this program is to identify physical and psychological dysfunctions left behind clinical recovery from SARS-CoV-2 infection. Participants to INTERCOP will be regularly followed-up in this program, having as an additional investigation a chest CT scan at 90 days, as per protocol.

### Outcomes {12}

#### Primary outcome

Time to negative conversion of SARS-CoV-2 nasopharyngeal swabs. Viral load will be measured by RT-PCR. [Time frame: baseline, days 3, 5, 7, 9, 11, 13, 15, 21, and 29]. IFNβ-1a has direct in vitro antiviral activity against SARS-CoV-2 [9], while IFNβ-1b has been identified as the key therapeutic reducing the time to negative conversion of SARS-CoV-2 nasopharyngeal swabs in a recent trial [11].

#### Secondary outcomes

- I)

- Improvement in clinical severity score, defined as percentage of patients reporting each severity rating on a 7-point ordinal scale (as defined above in the “Eligibility criteria {10}” section). [Time frame: baseline, days 7, 15, 21, and 29]. This study is underpowered to measure clinical efficacy. Nevertheless, measuring changes in the distribution of patients on the 7-point ordinal scale allows both to monitor the safety of the therapeutic regimen and detect potential positive changes in the clinical status of patients.
- Improvement in clinical severity score, defined as the time to clinical improvement of two points from the time of randomization on a 7-category ordinal scale or live discharge from the hospital, whichever comes first (as defined above in the “Eligibility criteria {10}” section). [Time frame: from baseline to day 29].
- Incidence of new oxygen use, non-invasive ventilation, or high flow oxygen devices during the trial. [Time frame: from baseline to day 29].
- Oxygenation free days in the first 28 days. [Time Frame: from baseline to day 29].
- Ventilator free days in the first 28 days. [Time frame: from baseline to day 29].
- Incidence of new mechanical ventilation use during the trial. [Time frame: from baseline to day 29].
- Number of patients transferred to Intensive Care Unit (ICU). [Time frame: from baseline to day 29].

- II) Mortality rate. [Time frame: from baseline to day 29].
- III) Changes from baseline in pulmonary CT imaging severity score measured with artificial intelligence and expressed as cc and percent values of diseased lung (lung consolidation, ground glass opacities and disease free) [Time frame: baseline, day 21; extra follow-up at 90 days].
- IV) Duration of hospital stay expressed in days. [Time frame: from baseline to day 29].
- V) Viral load measured on plasma with RT-PCR. [Time frame: baseline, days 3, 5, 7, 9, 11, 13, 15, 21, and 29].
- VI)

- Number of serious adverse events (SAE) and adverse drug reaction (expected and un-expected) until the discharge from the clinical unit (discharge for any motivation). [Time frame: from baseline to day 29]. This outcome addresses safety.
- Changes from baseline in white blood cell count (WBC), hemoglobin, platelets, C-reactive protein (CRP), lactate dehydrogenase (LDH), alanine aminotransferase (ALT), aspartate aminotransferase (AST), total bilirubin, creatinine, prothrombin time, international normalized ratio (INR), D-dimer, electrolytes (sodium, potassium, calcium), and glucose. [Time frame: baseline, days 7, 15, and 29]. IFNβ exerts broad-range effects on the immune system, being anemia, thrombocytopenia, and leukopenia reported as common events [21]. Thus, monitoring these markers, in conjunction with markers of inflammation, coagulation, and organ dysfunction, is of pivotal relevance to address safety.

#### Exploratory outcomes

- I)

- Cytokine and inflammatory profile changes from baseline (IL-6, ferritin, procalcitonin). [Time frame: baseline, days 7, 15, and 29]. Monitoring changes in markers of inflammation may help elucidate the complex and pleiotropic effects of IFN β-1a in vivo*.*
- White blood cell count (WBC), hemoglobin, platelets, CRP, LDH, prothrombin time, INR, D-dimer, glucose changes from baseline. [Time frame: baseline, days 7, 15, and 29]. In addition to addressing safety, these markers may help understand the effects of IFNβ-1a in vivo*.*

- II) Plasma and peripheral blood mononuclear cell mRNA expression profile of interferon-stimulated genes (ISG). Analysis of differentially expressed genes between the two groups. [Time frame: baseline, day 15]. This outcome may address whether route of administration and dose are adequate to obtain an effective immune response.
- III) Antibodies to SARS-CoV-2 measured by a newly developed luciferase immunoprecipitation system (LIPS) assay, measuring IgA, IgM, and IgG against RBD (receptor-binding domain RBD) and S (spike) proteins [Time frame: baseline, days 7, 15, and 29].
- IV) Antibodies to IFNβ-1a measured by a LIPS modification of a previously developed assay [22] [Time frame: baseline, days 7, 15, and 29]. A humoral response to IFNβ-1a is common among chronically treated patients with multiple sclerosis and may result in therapeutic resistance [23]. Whether a similar mechanism takes place in patients with COVID-19 treated with IFNβ-1a needs to be ascertained.

### Participant timeline {13}

**Fig. 1:**
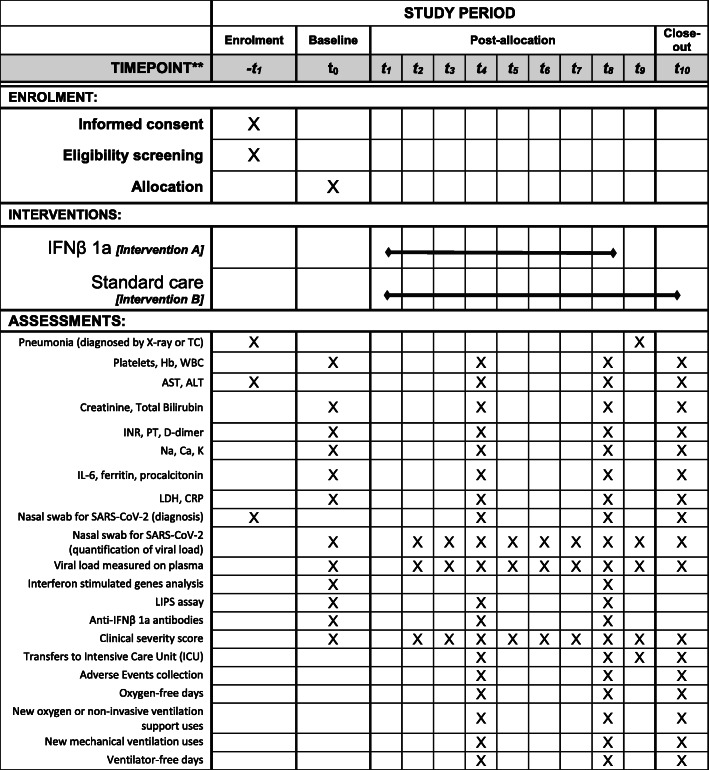
Schedule of enrolment, interventions, and assessments

The schedule of patient enrolment, interventions, and assessments is reported in Fig. 1.

**Fig. 2:**
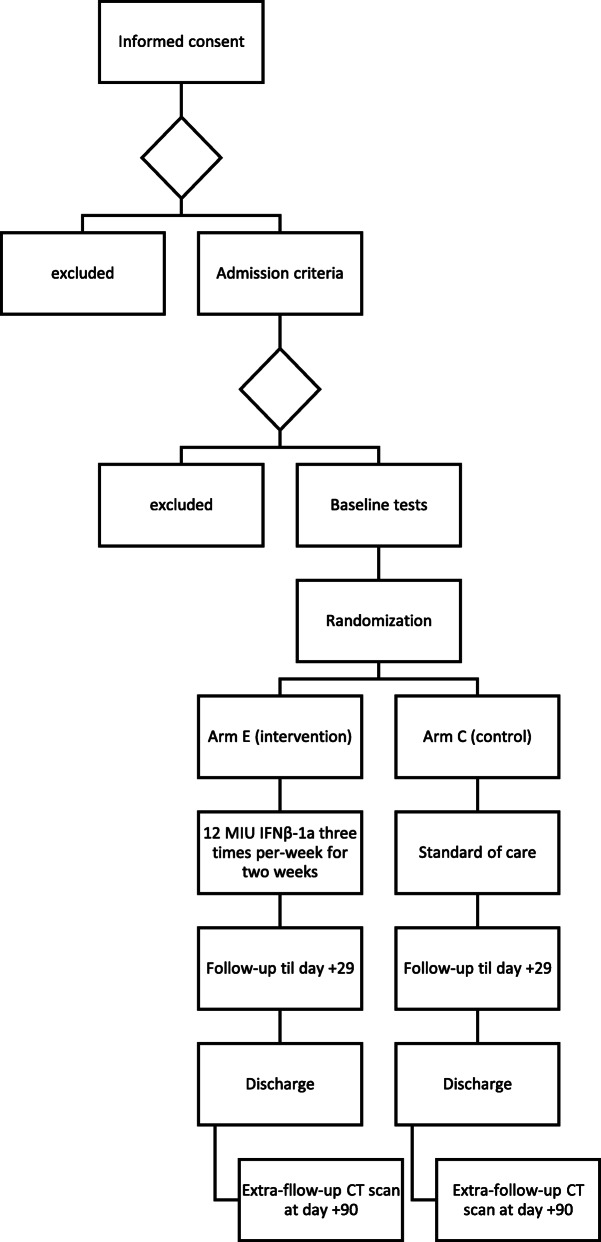
Study flow chart

The study flow chart is reported in Fig. 2

### Sample size {14}

The study is powered to assess the primary endpoint, which is time to negative conversion of nasal swabs for SARS-CoV-2. Based on the recent paper by Hung [11] and assuming a median time to negative conversion of nasal swabs for SARS-CoV-2 of 12 days in the standard of care group and 7 days in the IFNβ-1a group, to detect an HR of 0.58, with a power of 80% at 5% significance level, with an accrual duration of 6 months and a follow-up of 29 days, 120 total patients need to be randomized in 2:1 ratio. With a drop out of 5%, 126 patients will be accrued: 84 patients in the IFNβ-1a arm and 42 in the standard of care arm.

### Recruitment {15}

As stated above, San Raffaele Hospital is the largest clinical Hospital within the Gruppo San Donato (GSD) hospital network located in the Lombardy region, with a total capacity of more than 5000 beds. During the recent COVID-19 outbreak between February 23 and the end of May 2020, more than 1000 people diagnosed with COVID-19 were hospitalized at the San Raffaele Hospital and another 4000 in the remaining hospitals of the GSD network. Moreover, the San Raffaele Hospital has recently been identified as one of the reference hubs in the Lombardy Region. Therefore, in case of a second outbreak of COVID-19, the San Raffaele Hospital and the connected GSD network will easily have access to the diseased population and to patients suitable to trial recruitment.

### Assignment of interventions: allocation

#### Sequence generation {16a}

Patients will be randomized 2:1 to IFNβ-1a (active treatment) or standard of care (control arm), using a computer-generated list of random treatment allocations produced prior to the study start. The permuted block randomization method will be used. The block size will not be disclosed, to ensure concealment.

#### Concealment mechanism {16b}

Sequentially numbered, opaque, sealed envelopes will be used. Envelopes must be opened sequentially. To prevent subversion of the allocation sequence, the name and date of birth of the participant will be written on the envelope before opening it.

#### Implementation {16c}

The statistician will prepare the computer-generated list of random treatment and the staff of the Clinical Trial Center will provide sealed envelopes prior to the study start. The treating physician, who will open the sequentially numbered envelope, will randomize participants who meet all eligibility criteria on visit 2. For every randomization number, the corresponding code for the treatment group of the randomization list will be found inside the envelopes.

### Assignment of interventions: blinding

#### Who will be blinded {17a}

Trial participants, care providers, and data analysts will not be blinded.

#### Procedure for unblinding if needed {17b}

Not applicable, as there is no blinding.

### Data collection and management

#### Plans for assessment and collection of outcomes {18a}

Data will be collected and written in a case report form, (hereafter CRF) in paper format, by the data manager of the study, then will be stored in the principal investigator’s office, stored into the trial master file, including the source documentation reporting the patient’s data inserted in CRF, ready to be monitored by the CRA of the study. The source documentation reporting sensitive data, after the monitoring activity, will be anonymized, using the predefined study code for each patient.

#### Plans to promote participant retention and complete follow-up {18b}

There will be no specific plan for promoting participant retention and complete follow-up. Patients who discontinue or deviate will be followed up in the Outpatient Clinic and monitored for survival.

#### Data management {19}

After the monitoring activity, the data will be inserted in an Excel file, with restricted access (required password) stored on the institutional computer of the data manager (access requiring password, and automatic back-up in the main server), placed in his study, and, when planned in the protocol, the data will be transferred to the statistician, using an electronic support (flash drive), for the statistical analysis.

#### Confidentiality {27}

In accordance with European Regulation (EU) 2016/679 and current Italian Data Protection of Personal Authority, the collection and storage of personal health data will be limited to study-related information and will not be disclosed, made available, or otherwise used. All laboratory specimens, reports, data collection, process, and administrative forms will be identified by a coded ID number to maintain participant confidentiality. Only the physician and authorized personnel may be able to link this code to the name. All the information will be stored in locked file cabinets in areas with limited access. All local databases will be secured with password-protected access systems. Participant study information will not be released outside of the study without the written permission of the participant, except as necessary for monitoring by Ethics Committee, Italian and Foreign Regulatory Authorities.

#### Plans for collection, laboratory evaluation, and storage of biological specimens for genetic or molecular analysis in this trial/future use {33}

The collection and storage of biological material in the Biobank of the IRCCS Ospedale San Raffaele will be allowed only if participants accept the informed consent of COVID-BioB. Future research projects will always be submitted for approval to the Ethics Committee of IRCCS Ospedale San Raffaele.

### Statistical methods

#### Statistical methods for primary and secondary outcomes {20a}

Prior to the analysis of the final study data, a detailed statistical analysis plan (SAP) will be written describing all analyses that will be performed. Continuous variables will be described by mean, standard deviation, median, minimum, and maximum. Categorical variables will be described by number and percentage of subjects in each category. The primary efficacy analysis will be done on an intent to treat basis (ITT) and will include all randomized patients. Survival analysis will be performed to address the primary outcome of the study. Time to event is defined as the time from randomization until date of negative conversion. The date of the event (time of negative conversion) is the date of the first negative nasal swab test, confirmed by a second test performed at least 48 h apart. Data for dead patients are censored at the time of the last nasal swab. Survival curves will be estimated using the Kaplan-Meier method and the Log-rank test will be used to compare the outcomes of the different treatment arms. Hazard ratios will be estimated using the Cox proportional hazards model and these will be presented together with 95% two-sided confidence intervals. Endpoints that are measured as time from randomization will be compared between treatment groups using the Log-rank test. Student’s *t* test or Mann-Whitney test (depending on the data distribution pattern) will be used for inter-group comparison of continuous variables; chi-square test or Fisher exact test will be used for inter-group comparison of categorical variables. The primary analysis set for safety analyses is defined as the safety analysis set, which will include all participants who are randomized and have received at least 1 dose of study treatment. All safety data collected from the randomization date through the last follow-up visit will be summarized by treatment group. Statistical significance will be determined at 5% significance level. Analysis will be performed by SAS software Version 9.2 or later (SAS Institute, Inc., Cary, North Carolina, USA).

#### Interim analyses {21b}

No interim analyses are planned.

#### Methods for additional analyses (e.g., subgroup analyses) {20b}

No subgroup analyses are planned.

#### Methods in analysis to handle protocol non-adherence and any statistical methods to handle missing data {20c}

The primary efficacy analysis will be done on an intent to treat basis (ITT) and will include all randomized patient. A low drop-out rate is expected; therefore, missing data will not be replaced, and data analysis will be performed based on the observed cases principle.

#### Plans to give access to the full protocol, participant level-data, and statistical code {31c}

After end of the study, full protocol and data will be available upon request to the principal investigator.

### Oversight and monitoring

#### Composition of the coordinating center and trial steering committee {5d}

The coordinating center will include the PI, the co-investigator, the statistician, and the microbiologist; it will provide quality control, data collection, data analysis, and preparation of a report with the results.

There will be no steering committee because the study is monocentric and the coordinating center will take care of the integrity and quality of the data.

There will be no endpoint adjudication committee because the endpoints are straightforward and not subject to interpretation.

#### Composition of the data monitoring committee, its role and reporting structure {21a}

A data monitoring committee (DMC) is not planned because there are no expectations that the study drug will be harmful, the study endpoint(s) are clear, and the study duration is supposed to be brief.

A comprehensive quality control can be ordered in the form of monitoring. It might include checking the whole course of the study, management of documentation, management of data, management of enrolled patients, management of the experimental drug, and management of biological samples. The promoter of the study will be in charge for monitoring.

The investigators will assure the monitoring of the clinical study to assure conformance to protocol as well as the completeness, correctness, and plausibility of the completed case report forms (CRF).

#### Adverse event reporting and harms {22}

All adverse events (AEs) and adverse drug reactions (ADRs) regardless of seriousness or relationship to Medicinal Product or expectedness will be recorded daily on the corresponding page(s) included in the case report form (CRF). Whenever possible, symptoms will be grouped as a single syndrome or diagnosis. The investigator will specify the date of onset, maximal intensity, action taken with respect to the Medicinal Product, corrective therapy given, outcome, and his/her opinion as to whether there is a reasonable possibility that the event was caused by the study Medicinal Product.

Reports of serious AE (SAE) or serious ADRs, i.e., those events or reactions that result in death, life-threatening conditions, or prolongation of hospitalization, will be provided to the competent Authorities if requested.

The investigator will report all SAE immediately to the promoter and ethics committee. The investigator must fill the SAE report no later than 15 days. For reported deaths of a subject, the investigator shall supply the promoter and ethics committee with any additional information requested.

Once a year throughout the trial, the investigator will provide to competent regulatory authorities and the ethics committee an annual safety report, that is a list of all suspected serious ADRs which have occurred over this period and a report of the subjects’ safety.

#### Frequency and plans for auditing trial conduct {23}

If necessary, a comprehensive quality control could be ordered in the form of an audit. It might include checking the whole course of the study, the documentation, statistical analyses, and the investigators.

The promoter guarantees the availability for the inspections from the regulatory agencies.

#### Plans for communicating important protocol amendments to relevant parties (e.g., trial participants, ethical committees) {25}

Any modifications to the protocol that may impact on the conduct of the study, potential benefit of the patient or may affect patient safety, including changes of study objectives, study design, patient population, sample sizes, study procedures, or significant administrative aspects will require a formal amendment to the protocol. Such amendment will be approved by the ethics committee and notified to the Italian Regulatory Authority prior to implementation, in accordance with local regulations. Administrative changes, minor corrections, and/or clarifications of the protocol that have no effect on the study conduct will be notified to the ethics committee. All modifications will be tracked and specified also in the final report. Trial participants will be informed of any substantial modifications.

#### Dissemination plans {31a}

Since this is an academic clinical trial, there is not a dissemination plan other than the publication of results in scientific journals and meetings, when appropriate.

## Discussion

This trial addresses the current and urgent need to find an effective drug against SARS-CoV-2, the etiologic agent of the COVID-19 pandemic. In the absence of medications of proven efficacy, among drugs already available with other indications, we have identified INFβ, in its INFβ-1a clinical preparation variant, as an attractive candidate, due to a number of reasons [12]: (i) direct in vitro antiviral activity against SARS-CoV and MERS-CoV, reinforced by the evidence of specific anti-SARS-CoV-2 activity of INFβ-1a as recently demonstrated from our group [9]; (ii) previous encouraging experience in mice and nonhuman primate models of MERS [5, 6]; (iii) safety and tolerability in patients with ARDS, the most serious and threatening complication of COVID-19 [13]; and (iv) very promising results from a trial conducted in China, where INFβ was suggested to be the key component for the success of a combination treatment including INFβ, lopinavir–ritonavir, and ribavirin [11]. Moreover, INFβ-1a is an established treatment for multiple sclerosis, with long-term consolidated evidence of safety and tolerability [19]. Based on this ground, we designed a randomized, open-label controlled trial, investigating the efficacy and safety of IFNβ-1a in addition to standard of care vs standard of care alone in COVID-19 patients with mild-to-moderate pneumonia, with no confounding effects by other drugs specifically addressed to the etiological agent. An adequate sample size with a 2:1 randomization design, in conjunction with a primary outcome directly addressing the activity of the drug in terms of time to virus clearance, meets the need to gain insight about a relatively simple therapy, potentially accessible to large populations of patients in a rapid, adequate, and ethical way. Although this study might not detect significant changes in the distribution of patients within the seven-point ordinal scale, several biological (e.g., WBC, CRP, mRNA expression profiles) and clinical (e.g., pulmonary CT scan, oxygen-free days, number of transfers to ICU) parameters will be investigated as proxies for activity and efficacy.

## Trial status

Protocol version 0—May 14, 2020. Recruitment began in July 2020, and predicted completion of inclusion is in February 2021.

## Abbreviations

ADR: Adverse drug reaction
AE: Adverse event
COVID-19: Coronavirus disease
CRF: Case report form
ECMO: Extracorporeal membrane oxygenation
IgA: Immunoglobulin A
IgG: Immunoglobulin G
IgM: Immunoglobulin M
IL-6: Interleukin 6
ICU: Intensive care unit
IFNβ-1a: Interferon β-1a
LIPS: Luciferase immunoprecipitation system
mRNA: Messenger RNA
RBD: Receptor-binding domain
RT-PCR: Real-time PCR
SARS-CoV-2: Severe respiratory illness coronavirus 2
SAE: Serious adverse events
